# Bacterial deception of MAIT cells in a cloud of superantigen and cytokines

**DOI:** 10.1371/journal.pbio.2003167

**Published:** 2017-07-24

**Authors:** Johan K. Sandberg, Anna Norrby-Teglund, Edwin Leeansyah

**Affiliations:** 1 Center for Infectious Medicine, Department of Medicine, Karolinska Institutet, Karolinska University Hospital Huddinge, Stockholm, Sweden; 2 Program in Emerging Infectious Diseases, Duke-National University of Singapore Medical School, Singapore

## Abstract

The bacterium *Staphylococcus aureus* is an important cause of the life-threatening condition toxic shock syndrome in humans. Bacterial toxins known as superantigens (SAgs) generate this illness by acting as broad activators of a substantial fraction of all T lymphocytes, bypassing the normally highly stringent T-cell receptor antigen specificity to cause a systemic inflammatory cytokine storm in the host. In a new study, Shaler et al. found that immune cells called mucosa-associated invariant T (MAIT) cells make an unexpectedly large contribution to the SAg response in a largely T-cell receptor–independent, cytokine-driven manner. Subsequent to such activation, the MAIT cells remain unresponsive to stimulation with bacterial antigen. Thus, *S*. *aureus* hijacks MAIT cells in the cytokine storm and leaves them functionally impaired. This work provides new insight into the role of MAIT cells in antibacterial immunity and opens new avenues of investigation to understand and possibly treat bacterial toxic shock and sepsis.

T cells are lymphocytes that play critical and multifaceted roles in the immune defense of the human host. Situations where the T-cell compartment is compromised and functions poorly or in a misguided fashion often lead to very serious conditions ranging from immunodeficiency to autoimmunity and immunopathology. The majority of T cells respond in an adaptive fashion to peptide antigens derived from pathogen proteins and this recognition is governed by major histocompatibility complex (MHC)-encoded antigen-presenting molecules. However, the T-cell compartment also includes several types of unconventional T-cell subsets that recognize antigens presented by nonclassical MHC-like molecules [1]. Mucosa-associated invariant T (MAIT) cells are a subset of such unconventional T cells that have recently gained considerable attention (Box 1). MAIT cells exist in expanded numbers in adult humans and have a mature phenotype ready to respond to antigens. The differentiation and maturation of MAIT cells can be observed already in fetal tissues [2], while the expansion to 1%–10% of T cells in peripheral blood occurs after birth [3].

**Box 1. What are MAIT cells?**

In humans, mucosa-associated invariant T (MAIT) cells are classically defined by their semi-invariant T-cell receptor (TCR) containing the invariant TCR α-chain variable region 1–2 (TRAV1-2 or commonly known as Vα7.2) coupled with TCR α-chain joining region 12, 20, or 33 (TRAJ12, 20, or 33 or Jα12, 20, 33), and a restricted TCR β-chain repertoire, namely, TCR β-chain variable region 6 (TRBV6 or Vβ13) and TRBV20 (Vβ2) [4–6]. The concomitant expression of their unique semi-invariant TCR with high levels of the C-type lectin receptor CD161 or the IL-18 receptor α subunit (IL-18Rα) identifies the MAIT cell population in blood and tissues [7,8]. The majority of human MAIT cells express the CD8 coreceptor, with a minor population expressing neither CD4 nor CD8 and only a minute population expressing CD4 [7,8]. MAIT cells express the tissue-homing and inflammatory-chemokine receptors, such as CCR5, CCR6, CCR9, CXCR3, CXCR4, and CXCR6 [3,9], allowing migration to both mucosal and other tissues as well as to sites of inflammation. Consequently, MAIT cells are found in high abundance in peripheral blood, the intestines, lungs, and liver of healthy humans [3,8]. Recent discoveries showed that MAIT cells recognize microbial riboflavin metabolites presented by the major histocompatibility complex (MHC) class-Ib related protein 1 (MR1) [10,11]. The critical and conserved nature of microbial riboflavin synthesis allows MAIT cells to respond to a wide range of microbes, including both pathogens and commensals alike [8] (see Box 2). Because MR1 also displays an extraordinary level of evolutionary conservation among placental and marsupial mammals [12,13], it is not surprising that MAIT cells have been found in a variety of mammals to date, including primates, rodents, and ruminants [12,14,15]. Interestingly, MHC-Ib-restricted T cells’ evolutionary conservation extends to the amphibian *Xenopus* spp., where invariant T cells are a critical component of early antiviral immunity [16]. MAIT cells develop in the thymus and follow several developmental stages under the control of multiple key factors, including the restriction element MR1, the transcription factor promyelocytic leukaemia zinc finger (PLZF, also known as zinc finger and BTB domain-containing protein 16 [ZBTB16]), and possibly microbial colonization [2,7,17]. The transcription factor PLZF is critical for MAIT cell maturation and functionality, a universal feature of the innate-like T-cell lineages [18]. Interestingly, while maturation of MAIT cells in mice requires the establishment of the gut microbiota [5,7], human fetal MAIT cells are already functionally mature in the mucosal tissues devoid of such established microbial colonization [2]. In summary, the abundance of MAIT cells, the highly conserved nature of MR1 across mammals, and equally conserved nature of the MAIT cell antigens across microbial species strongly suggest that this population of innate-like T cells plays important roles in protection of the host.

MAIT cells carry a T-cell receptor (TCR), which has conserved and invariant features. Using this TCR, they recognize antigens in complex with the MHC-Ib-related protein 1 (MR1) [5]. MR1 displays an extraordinary level of evolutionary conservation among placental and marsupial mammals [12,13], strongly supportive of the notion that MR1 and MAIT cells perform critical functions in the immune system. The nature of MR1-presented antigens was long elusive. In 2012, it was discovered that MAIT cells recognize microbial vitamin B_2_ (riboflavin) metabolites from a wide range of microbes presented by MR1 molecules [10] (Box 2) (Fig 1A). The conserved nature of this pathway underlies the ability of MAIT cells to respond to a diverse range of microbes, including *Escherichia coli*, *Mycobacterium tuberculosis*, *Candida albicans*, *and S*. *aureus*. Once activated by an antigen, MAIT cells rapidly produce pro-inflammatory cytokines, including interferon-gamma (IFNγ), tumor necrosis factor (TNF), and interleukin-17 (IL-17) [8,19]. This mixed cytokine profile most probably contributes to the reported role of MAIT cells in the protection against bacterial and mycobacterial lung infections in animal models as well as in human pulmonary tuberculosis [8,20–23].

**Box 2. Antigenic vitamin B metabolites presented by major histocompatibility complex class-Ib related protein 1 (MR1)**

Two recent seminal studies discovered that MR1 molecules bind and present vitamin B_2_ (riboflavin) and vitamin B_9_ (folic acid) metabolites [10,11]. Interestingly, the vast majority of MAIT cells are reactive only to metabolic intermediates and derivatives of microbial riboflavin biosynthesis. Consistent with these findings, a key distinction between the microbes that are stimulatory and nonstimulatory to MAIT cells is that the former synthesize riboflavin, whereas the latter do not [8,10]. Riboflavin is a critical component of the cofactors flavin adenine dinucleotide and flavin mononucleotide, key players in a wide variety of microbial cellular processes, including redox reactions, energy metabolism, and biosynthesis of many macromolecules. MAIT cell activation requires key genes encoding critical enzymes, including *ribA* and *ribD*, that form 5-amino-6-ribityl aminouracil (5-A-RU), an early aminopyrimidine intermediate in riboflavin biosynthesis [11]. 5-A-RU then gives rise to MAIT cell–activating antigens by the nonenzymatic condensation with host- or bacterial-derived small-molecule metabolic intermediates, such as glyoxal and methylglyoxal [11]. These otherwise unstable aminopyrimidine antigens are subsequently “captured” by MR1 and stabilized as the antigen-MR1 complex within the host cells’ endoplasmic reticulum before translocating to the plasma membrane, leading to presentation at the cell surface and recognition by MAIT cells [24].

**Fig 1:**
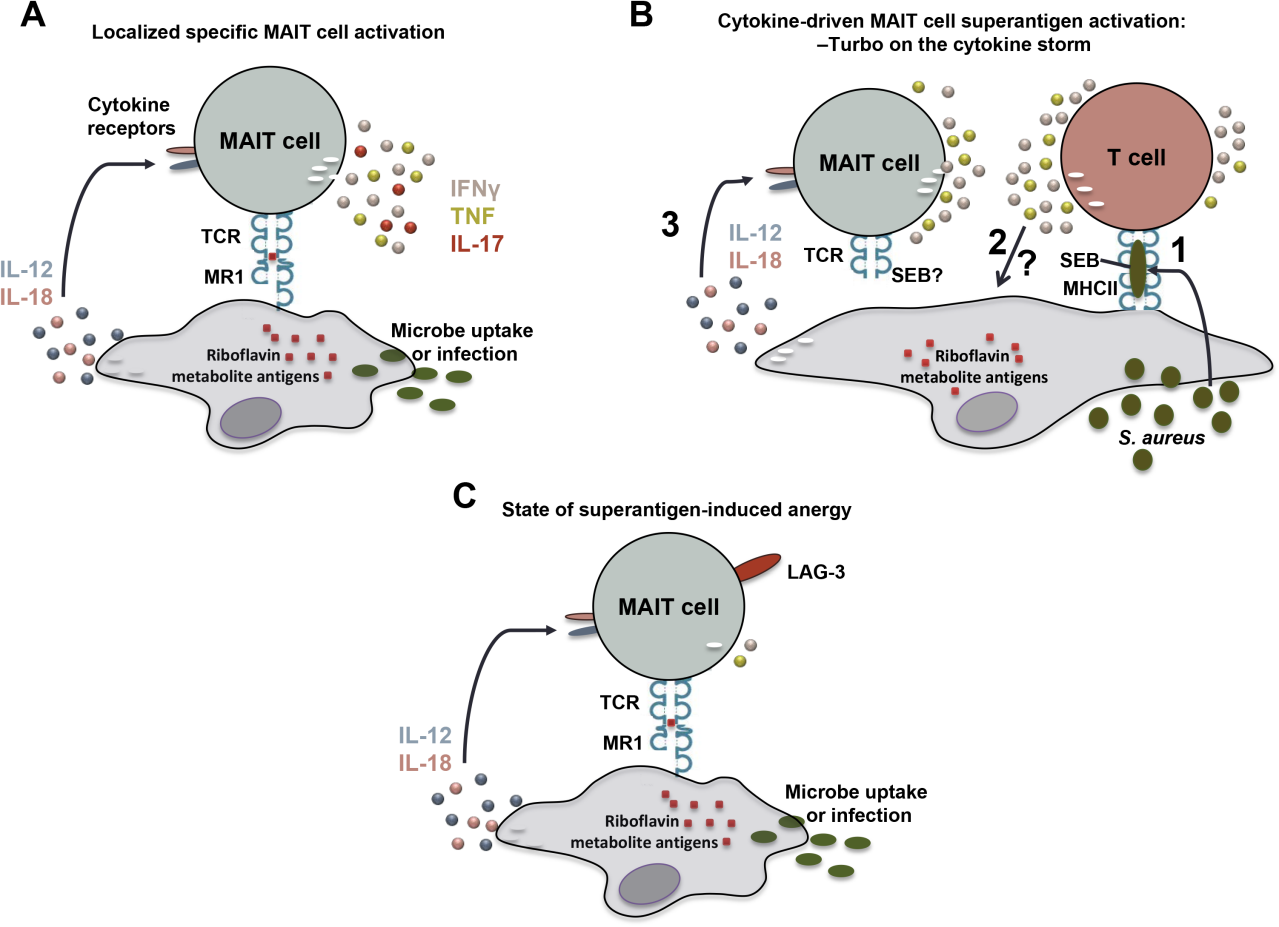
Deception and diversion of MAIT cell responses by bacterial superantigen. **(A)** Mucosa-associated invariant T (MAIT) cells recognize bacterial riboflavin metabolites presented by major histocompatibility complex class-Ib related protein 1 (MR1) molecules. This response is enhanced by interleukin 12 (IL-12) and interleukin 18 (IL-18) produced by the antigen-presenting cell. **(B)** Staphylococcal enterotoxin B (SEB) activates a cytokine storm by conventional T cells, which in turn strongly activates MAIT cells. This MAIT-cell response enhances the already ongoing cytokine storm. There is also some contribution of direct SEB effects on a small fraction of MAIT cells via their T-cell receptor (TCR). **(C)** After the cytokine storm, MAIT cells become hyporesponsive and this anergic state depends at least partly on the inhibitory receptor lymphocyte-activation gene 3 (LAG-3).

In addition to their capacity for TCR-mediated recognition of microbial riboflavin metabolites, MAIT cells also express a range of receptors usually associated with so-called “innate” immune cells including natural killer (NK) cells [25]. This includes expression of receptors for ILs that can mediate “innate-like” activation of immune cells, including IL-18 and IL-12. Interestingly, Ussher et al. showed that MAIT cells as well as other T cells sharing the marker CD161 can be activated to produce IFNγ independently of the TCR by stimulation with IL-12 and IL-18 [26]. This finding broadened the potential role of MAIT cells in immune responses beyond infections caused by microbes carrying the riboflavin biosynthesis pathway to also include other situations where IL-12 and IL-18 might be produced. This cytokine mode of activation may be a useful tool for the immune system in the context of viral infection where IL-12 and IL-18 are induced, and MAIT cell production of IFNγ in the local milieu can have direct antiviral effects. Indeed, activation of MAIT cells was recently shown to occur in several different types of human viral infections [27]. In this viral context, MAIT cell activation was dependent on IL-18 in synergy with IL-12, IL-15, and interferons, and the IFNγ produced was able to inhibit hepatitis C virus replication in vitro, suggesting direct disease relevance. Thus, in the setting of acute viral infection, MAIT cells recruited to the site of infection may in this way act as amplifiers of the innate antiviral response.

Strong and largely nonspecific activation of an immune cell subset such as MAIT cells may, however, also pose severe danger to the host, in particular since MAIT cells represent up to 10% of T cells in blood and even up to 35% in liver and some mucosal sites [3,5,12,28]. In the June issue of *PLOS Biology*, Shaler et al. presents evidence that this mode of MAIT cell activation can be triggered by the superantigen (SAg) staphylococcal enterotoxin B (SEB) and that MAIT cells represent a substantial source of pro-inflammatory cytokines following SAg exposure, suggesting a role in staphylococcal immunopathology and immunosuppression [29]. SAgs are potent bacterial exotoxins secreted by bacteria including *S*. *aureus* and *Streptococcus pyogenes*, which cause local symptoms such as food poisoning as well as systemic life-threatening conditions such as toxic shock syndrome (TSS; Box 3) [30]. SAgs can do this because they act as oligoclonal activators of significant parts of the T-cell repertoire, where the fine MHC-peptide specificity of T cells is bypassed when the SAg cross-links MHC class II molecules on antigen-presenting cells and TCRs on T cells. This leads to TCR signalling and massive T-cell activation with concomitant release of inflammatory mediators. However, the interaction between SAgs and TCRs is not completely indiscriminate as only certain TCR β-chain variable (Vβ) segments can be engaged in this way, leaving only a minority of T cells susceptible to triggering by any given SAg. Shaler et al. dissected the human T-cell response to SEB, one of the main SAgs produced by *S*. *aureus*, and found that MAIT cells make an oversized contribution to the IFNγ response against SEB relative to their frequency among T cells. This MAIT cell response was dependent on MHC class II but not on MR1 and the magnitude of the response was surprising given that only a small minority of MAIT cells normally express TCR Vβ segments that would bind SEB. In subsequent experiments, Shaler et al. found that this strong MAIT cell activation and IFNγ production was instead primarily driven by IL-12 and IL-18 produced by other cells among peripheral blood mononuclear cells as a result of the SEB-mediated activation of polyclonal T cells (Fig 1B). Both IL-12 and IL-18 were required for full activation and inhibitors of components of the mitogen-activated protein (MAP) kinase pathway were able to block this MAIT cell response. The need for IL-12 and MHC class II in this cytokine-driven mode of activation indicates that so-called professional antigen-presenting cells, such as dendritic cells, initiate this MAIT cell response.

**Box 3. Superantigens and their role during gram-positive bacterial infections**

The gram-positive bacteria *Staphylococcus aureus* and *Streptococcus pyogenes* are important causes of life-threatening toxic shock syndrome (TSS), a fulminant systemic disease characterized by fever, hypotension, and multiorgan dysfunction. Key mediators of TSS are streptococcal and staphylococcal exotoxins that belong to the family of superantigens (SAgs) (reviewed in [31]). In *S*. *pyogenes* and *S*. *aureus*, 11 and 23 genetically distinct SAgs have been identified, respectively, and most clinical isolates secrete at least 1 SAg [32]. SAgs are defined by their ability to interact with and activate innate immune cells and T cells in an unconventional manner, bypassing normal rules for antigen processing and presentation. They bind directly to major histocompatibility complex class II molecules without prior cellular processing and outside the antigen-binding cleft as well as to specific Vβ regions of the T-cell receptor [30]. This leads to massive T-cell activation and proliferation and consequently a cytokine storm resulting in systemic toxicity and shock. Although the SAg contribution to disease pathophysiology is undeniable, the question that has puzzled the scientific field since their discovery is how they influence bacterial fitness and survival. Many studies have indicated that the SAg-triggered overwhelming cytokine storm results in an unbalanced and, hence, dysfunctional immune response (reviewed in [33]). More recently, it was also demonstrated that SAgs are critical for the bacterial life cycle. Kasper et al. demonstrated that SAgs supported bacterial colonization of nasal mucosa and thereby the initial stages of infection [34]. Furthermore, a role in bacterial survival could be linked to SAg-triggered neutrophil chemotaxis and consequent abscess formation in the liver [35]. This shows the versatility of SAgs influencing a broad range of immune cells and promoting bacterial infections during different stages of infections.

These findings may at first glance not make total sense. Why would an efficient colonizer and pathogen such as *S*. *aureus* keep a toxin which not only triggers a broad T-cell response but also generates a secondary cytokine-driven activation of MAIT cells? This is especially intriguing given that *S*. *aureus* has the riboflavin biosynthesis pathway and generates the MR1- presented antigens that MAIT cells can detect [8]. Here, the paper by Shaler et al. provides a possible answer with their finding that SEB-stimulated hyperactivation of MAIT cells renders them unresponsive to a subsequent encounter with an MR1-presented bacterial antigen (Fig 1C). This state of cellular unresponsiveness after activation is often referred to as “anergy.” In this system, anergy was associated with the induction of the inhibitory receptors known as T cell immunoglobulin and mucin-3 (TIM-3) and lymphocyte-activation gene 3 (LAG-3) on MAIT cells, and blocking of LAG-3 could restore their ability to respond to bacteria. Finally, the up-regulation of these inhibitory receptors on MAIT cells was also seen in vivo in humanized mice injected with SEB. Altogether, these findings suggest that these effects may be a deception and diversion strategy by *S*. *aureus* to avoid direct recognition by MAIT cells.

The IL-12– and IL-18–mediated activation of MAIT cells is likely to have desired consequences during, for example, a local virus infection, in which enhanced IFNγ production can help limit virus replication, as discussed above. In the context of *S*. *aureus* infection, this mechanism appears to have been hijacked as a diversion mechanism that anergizes the MAIT cells and makes them unable to respond to MR1-restricted antigen. This way, the bacterium may avoid direct recognition, with detrimental consequences for host antibacterial immunity, not only against *S*. *aureus* but also against many other microbial infections. A potentially even more serious consequence for the host may occur in the context of systemic spread of bacterial toxin SAgs, in which the MAIT-cell contribution to the cytokine storm may provide a turbo effect and increase the risk of shock and systemic toxicity. This is a life-threatening condition and the realization that MAIT cells contribute to the SAg-triggered cytokine storm may open new possible avenues for patient treatment.

The work by Shaler et al. provides significant new insight into the role of MAIT cells in the complex interaction between a bacterial pathogen and the host. Still, many open questions remain regarding the specific *S*. *aureus* SAg effects on MAIT cells and the downstream consequences, as well as broader questions concerning other SAg-triggered diseases and how MAIT cell contribution to antibacterial immunity may be affected. For example, the perspective of time may be very important here; what is the relative contribution of TCR Vβ-dependent and cytokine-dependent MAIT cell activation during various stages of activation and during bacterial encounter? To what extent will the induced anergy prevent MAIT cell recognition of different bacteria? Will the MAIT cell anergy be local or systemic, and how long will it last? Will these effects limit immune defenses to subsequent encounters of unrelated bacterial, fungal, or viral pathogens? Can pharmaceutical intervention be designed to limit MAIT cell activation in this setting to prevent the immunopathology and the downstream effects? The answers to questions such as these will be very important to help understand the complex relationship between MAIT cells and bacterial pathogens and help define the role that these cells play in host protection.

## Abbreviations

5-A-RU: 5-amino-6-ribityl aminouracil
IFNγ: interferon-gamma
IL: interleukin
LAG-3: lymphocyte-activation gene 3
MAIT: mucosa-associated invariant T
MAP: mitogen-activated protein
MHC: major histocompatibility complex
MR1: MHC class-Ib related protein 1
NK: natural killer
PLZF: promyelocytic leukaemia zinc finger
SAg: superantigen
SEB: staphylococcal enterotoxin B
TCR: T-cell receptor
TIM-3: T cell immunoglobulin and mucin-3
TNF: tumor necrosis factor
TRAJ: TCR α-chain joining region
TRAV: TCR α-chain variable region
TRBV: TCR β-chain variable region
TSS: toxic shock syndrome
Vβ: β-chain variable
ZBTB16: zinc finger and BTB domain-containing protein 16
